# Deciphering the Underlying Mechanism of Eucommiae Cortex against Osteoporotic Fracture by Network Pharmacology

**DOI:** 10.1155/2020/7049812

**Published:** 2020-09-07

**Authors:** Yongming Shuai, Zhili Jiang, Qiuwen Yuan, Shuqiang Tu, Fanhui Zeng

**Affiliations:** Nanchang Hongdu Hospital of Traditional Chinese Medicine, 264 Minde Road, Donghu District, Nanchang, Jiangxi 330006, China

## Abstract

**Background:**

Du Zhong (DZ), or Eucommiae Cortex, a traditional Chinese herbal medicine, has been used to treat osteoporosis. Although it has been reported that DZ can improve bone mass in ovariectomized rats, its pharmacological mechanisms in treating osteoporotic fractures (OPF) remain unclear.

**Methods:**

In this study, we used a network pharmacological manner to explore its potential complicated mechanism in treating OPF. We obtained DZ compounds from TCMSP and BATMAN-TCM databases and collected potential targets of these compounds through target fishing based on TCMSP and BATMAN-TCM databases. Next, we collected the OPF targets by using CTD, GeneCards, OMIM, HPO, and GenCLiP 3 databases. And then the overlapping genes between DZ potential targets and OPF targets were used to build up the protein-protein interaction (PPI) network and to analyze their interactions and find out the big hub genes in this network. Subsequently, clusterProfiler package in *R* language was utilized to conduct the enrichment of Gene Ontology biological process and KEGG pathways.

**Results:**

There were totally 93 active compounds and 916 related targets in DZ. After the enrichment analysis, we collected top 25 cellular biological processes and top 25 pathways based on the adjusted *P* value and found that the DZ anti-OPF effect was mainly associated with the regulation of ROS and inflammatory response. Furthermore, 64 hub genes in PPI network, such as MAPK1 (degree = 41), SRC (degree = 39), PIK3R1 (degree = 36), VEGFA (degree = 31), TP53 (degree = 29), EGFR (degree = 29), JUN (degree = 29), AGT (degree = 29), MAPK1, SRC, PIK3R1, VEGFA, and TP53, were considered as potential therapeutic targets, implying the underlying mechanisms of DZ acting on OPF.

**Conclusion:**

We investigated the possible therapeutic mechanisms of DZ from a systemic perspective. These key targets and pathways provided promising directions for the future research to reveal the exact regulating mechanisms of DZ in treating OPF.

## 1. Introduction

Osteoporosis is a systemic bone disorder characterized by low bone density, poor bone quality, reduced bone strength, and an accompanying increased incidence of fractures [1]. The latest study noted that over 10 million people suffer from osteoporosis in the United States. Meanwhile, the treatment burden of osteoporosis, $22 billion in 2008, is expected to rise due to the consistently increasing aging population. Related osteoporosis treatments should be used to alleviate or reduce symptoms such as fractures, which could result in considerable cost savings [2]. Osteoporotic fractures (OPF) have serious consequences, such as declining functions and independence as well as increased morbidity and mortality [3, 4]. Hip fractures are considered to be the most expensive and debilitating of fractures because approximately 10 to 20% of patients will end up with a disability in the year following a hip fracture and half of patients will lose their independence. Osteoporotic fractures are also associated with motor function decline when occurring at other skeletal sites [5]. To curb bone loss, maintain bone mass, and decrease the risk of OPF, antiresorptive agents, anabolic agents, and bone mineral drugs have been widely used in clinical treatment [6]. However, it is noteworthy that almost all these antiosteoporosis treatments have limitations and side effects, such as increasing the risk of breast cancer, jaw necrosis, or atypical femur fracture [7, 8]. Therefore, alternative and safe intervention strategies may be promising.

Recently, traditional Chinese medicine (TCM) has attracted worldwide attention and has served as a main alternative treatment in East Asia, North America, and Europe due to its satisfactory curative effect, relatively low toxicity, and low cost [9–11]. TCM has been used in China for thousands of years, and it exerts a beneficial therapeutic effect and reduces side effects through multiple herb combinations to prevent and treat various diseases [12]. Meanwhile, TCM has increasingly been found to be effective in the treatment of osteoporosis [13, 14].

Du Zhong (DZ), or Eucommiae Cortex, is one of the most commonly used TCM herbs in the treatment of osteoporosis. DZ has been found to facilitate osteogenesis through activating osteoblasts and to inhibit osteolysis by suppressing osteoclast activity [15]. An animal experiment confirmed that Du Zhong Wan, consisting of Eucommiae Cortex and Radix Dipsaci, is capable of improving trabecular bone mineral density and bone biomechanical properties and thus plays a key role in the treatment of osteoporosis [16]. Although DZ exerted therapeutic effects against osteoporosis, the therapeutic mechanism of its exact is still unclear. Similar to TCM, DZ exerts its therapeutic efficacy by regulating multiple molecules in the human body. Therefore, it is still a major challenge to prove the mechanism of DZ through scientific trials that are used in Western medicine. Fortunately, the development of systems pharmacology offers researchers an alternative opportunity and an option to investigate the pharmacological mechanisms of TCM [17]. Recently, Wang et al. utilized a network pharmacology method to clarify the synergistic mechanism of Er-Xian decoction in treating osteoporosis [18]. Similarly, Gan et al. employed this systems pharmacology method to dissect the mechanisms of the therapeutic effect of Rhizoma Drynariae on osteoporosis [19].

**Figure 1:**
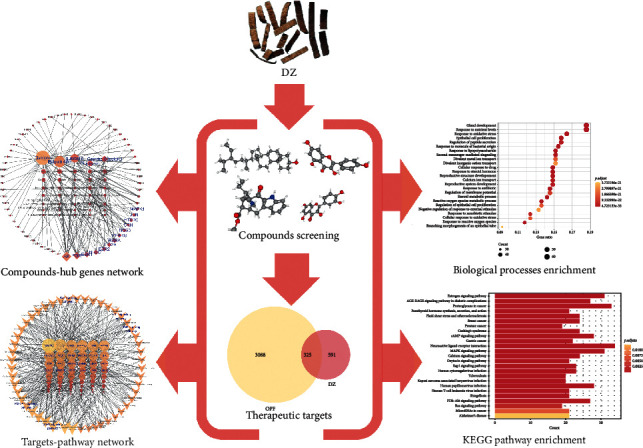
The schematic map of the present study to investigate potential mechanisms of DZ in the OPF treatment.

In this network pharmacology work, we aim to comprehensively dissect the mechanisms of DZ in treating OPF. We collected related compounds of DZ from multiple databases and obtained the compounds' potential targets via target fishing. Then, we matched these targets with OPF-related targets that were collected from a multisource database. Next, using overlapping targets obtained from the previous process, we built a protein-protein network to analyze their internal interactions and then screened out the hub genes. Furthermore, we used the clusterProfiler package in *R* to conduct biological process and KEGG pathway enrichment analyses. The protocol of our experimental procedures is shown in Figure 1.

## 2. Materials and Methods

### 2.1. Identification of Chemical Ingredients

To collect the active compounds of DZ, we utilized the Traditional Chinese Medicine System Pharmacology Database (TCMSP™, http://lsp.nwu.edu.cn/tcmsp.php) [20], which is a frequently used platform for systems pharmacology. The BATMAN-TCM platform (http://bionet.ncpsb.org/batman-tcm/) [21], one of the largest comprehensive TCM platforms, uses the similarity of known TCM drug–target interactions to predict potential compound–target interactions. In total, ninety-three chemical compounds were collected in this part.

### 2.2. Compound Screening and Target Prediction

#### 2.2.1. OB Evaluation

Oral bioavailability (OB) refers to the percentage of an orally administered drug that reaches systemic circulation, and it is one of the most important pharmacokinetic profiles for drug screening. The TCMSP platform has adopted the OBioavail1.1 system, which integrates P450, 3A4, and P-glycoprotein information to obtain the OB value [22]. To determine the active ingredients used for further steps, we set the OB threshold at 30%.

#### 2.2.2. DL Prediction

Drug-likeness (DL) is a qualitative index that represents the degree to which the target compound is “drug-like” and is used to remove chemically unsuitable compounds. TCMSP uses the Tanimoto similarity method to calculate the DL index by comparing the target compound to all 6511 molecules in the DrugBank database [23]. In this process, the compounds that did not meet the condition that DL ≥ 0.18 were removed.

#### 2.2.3. BATMAN-TCM

To collect candidate compounds, we set the potential TAR score cutoff ≥20 and *P*-value cutoff <0.05.

#### 2.2.4. Target Prediction of the Active Ingredients

The compounds have effects on the targets that induce them to exert their biological functions. Thus, we used the TCMSP and BATMAN-TCM platforms to predict the targets of active compounds.

### 2.3. Disease Target Identification by Multiple Databases

We used multiple databases to collect OPF-related targets, and the terms “Osteoporotic” and “Fracture” were used as the key words for the search. The databases in this step included the Comparative Toxicogenomics Database (CTD, http://ctdbase.org/) [24], GeneCards (https://www.genecards.org/) [25], OMIM (https://www.omim.org/) [26], HPO (https://hpo.jax.org/app/) [27], and GenCLiP3 (http://ci.smu.edu.cn/genclip3/analysis.php) [28]. In total, three thousand four hundred-thirteen OPF targets were found through these databases. Then, we constructed a Venn diagram to determine the overlap between OPF and the active compound targets because these overlapping targets may play a critical role in the treatment of OPF. These targets were analyzed by String (https://string-db.org/) [29], in which the minimum required interaction score was set at the highest confidence level (0.900), and then, the protein-protein interaction (PPI) data were exported.

### 2.4. Network Construction

#### 2.4.1. Construction Method

Two main networks were built in this process, including the compound-hub gene network and the hub gene-pathway network. The target information was obtained from the KEGG pathway enrichment results. Cytoscape 3.6.2 (http://www.cytoscape.org/) was used for all the network visualization work. Cytoscape 3.6.2 is one of most powerful open-source software programs for constructing different networks visually [30]. However, there are several limitations faced by network pharmacology research. For example, questions including whether compounds activate or inhibit targets, how they exact their effects on targets, whether they use binding or catalysis, and so on remain unclear.

#### 2.4.2. Topological Feature Definition

We used three parameters to describe and quantify the importance of nodes in these networks because nodes that bridge many edges with their neighbors are more likely to exert crucial mediating functions. (1) “Degree” means the number of edges shared with other nodes. Examining the node's degree is the most straightforward method of quantifying its centrality [31]. (2) “Betweenness centrality” is used to measure node centrality based on the shortest paths. A high-value node would play a more important role in the network because more regulation information will pass through it [32]. (3) “Closeness centrality” represents the mean distance between the node and all the other nodes in the network and is the reciprocal of the sum of the length of the shortest paths between a node and all the other nodes in the network. Therefore, the central node would be more likely to be close with all other nodes [33].

### 2.5. Biological Process and Pathway Enrichment Analysis

We used the clusterProfiler package in *R* (*R* 3.6.2 for Windows) to perform Gene Ontology (GO) enrichment analysis and Kyoto Encyclopedia of Genes and Genomes (KEGG) pathway analysis to identify biological processes and systemic involvement of pathways.

## 3. Results

### 3.1. Active Compounds of DZ

A total of 147 related compounds were found in DZ collected from the TCMSP database, and of these, 27 candidate compounds remained after the screening of ADME thresholds (OB ≥ 30%, DL ≥ 0.18). Using the BATMAN-TCM database, we obtained 73 active compounds that matched the filter criteria. In total, we collected 93 unique compounds.

### 3.2. Target Prediction and Analysis

**Figure 2:**
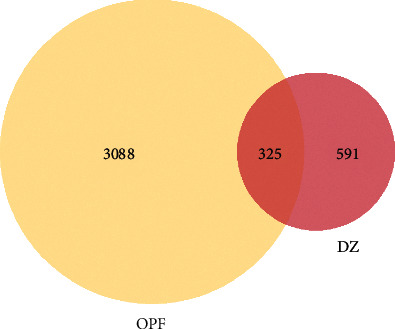
The Venn diagram for the targets of DZ and OPF. The overlap targets are the potential therapeutic genes for DZ when treating OPF.

For these unique compounds, we obtained 961 unique related targets, 104 from TCMSP and 857 from BATMAN-TCM. We integrated the OPF genes that were obtained from multisource databases, including the CTD, GeneCards, OMIM, HPO, GenCLiP 3 databases, and a total of 3,834 related genes were collected. After construction of the Venn diagram, three hundred twenty-five overlapping targets between the related targets of DZ and OPF were selected as the key targets on which DZ exerts its anti-OPF effects (Figure 2).

**Figure 3:**
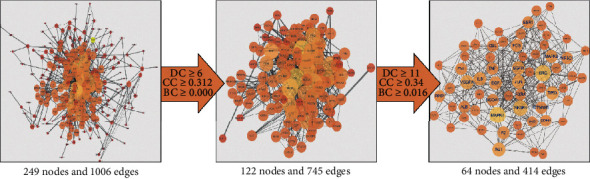
The whole screening process for the PPI network through a topological method. In these images, the bigger size and brighter color represent higher DC value.

The data obtained from the String database were used to establish the PPI network for the 325 overlapping targets. In this network, there were 249 nodes and 1006 edges in total. Then, three main parameters, namely, “degree”, “betweenness”, and “closeness”, were used as filters to select key genes and to build the large hub nodes to determine the anti-OPF effect of DZ. The first screening threshold was degree ≥ 6, closeness ≥ 0.312, and betweenness ≥ 0.000, which resulted in 122 nodes and 745 edges. Then, these 72 key nodes were further screened with a second threshold consisting of degree ≥ 11, closeness ≥ 0.340, and betweenness ≥ 0.016, and 64 nodes and 414 edges remained after this screening (Figure 3). These nodes included MAPK1, SRC, PIK3R1, VEGFA, TP53, EGFR, JUN, AGT, IL6, EGF, MAPK8, FOS, F2, FN1, EDN1, TNF, PPBP, ESR1, NCOA1, CTNNB1, NR3C1, ALB, RXRA, CBL, INS, PRKACA, AHSG, TGFB1, HSPA8, AR, FGF23, GNAS, NFKB1, GAS6, PRKCD, AVPR2, APOA1, PDGFB, HSP90AB1, IL4, HSPA1A, IGF2, SIRT1, PPARG, PPARA, CDC42, PGR, IFNG, CST3, NCOA3, TAC1, PLCB1, NOS3, CCL5, FGF2, ESR2, STAT5A, WNT5A, HTR2C, HTR2A, CCL2, CRH, APOE, and CFD (Table 1). After sorting these 64 big hub nodes in descending order, we found that MAPK1 (degree = 41), SRC (degree = 39), PIK3R1 (degree = 36), VEGFA (degree = 31), TP53 (degree = 29), EGFR (degree = 29), JUN (degree = 29), AGT (degree = 29), IL6 (degree = 28), EGF (degree = 27), MAPK8 (degree = 26), FOS (degree = 25), and F2 (degree = 25) were the most important targets in this PPI network.

**Figure 4:**
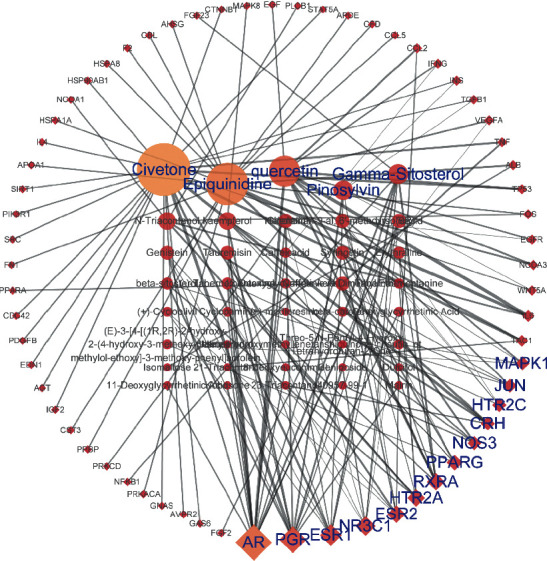
The network for big hub genes-compounds connection. The diamond shape nodes are the hub genes and round ones represent compounds. And all nodes' color changes according to their degree value.

Then, we further built the big hub nodes-compound network (Figure 4) based on these 64 key targets. This network included 40 compound nodes and 64 large hub target nodes. Subsequently, we reordered these compound nodes in descending order of degree and found that Civetone was relevant to 25 large hub genes, Epiquinidine to 19 genes, quercetin to 13 genes, Gamma-Sitosterol to 7 genes, and Pinosylvin to 7 genes.

### 3.3. GO Biological Process Enrichment Analysis

**Figure 5:**
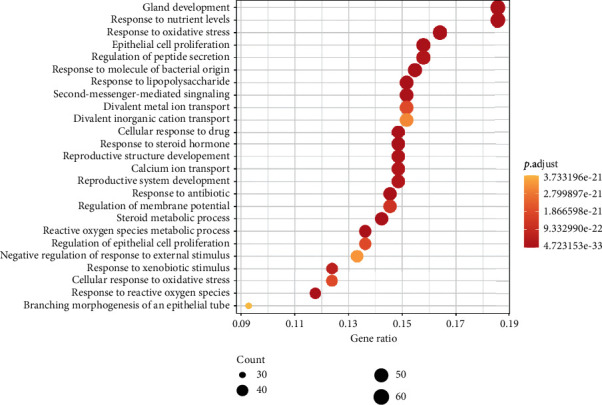
Top 25 processes of the biological process enrichment.

After sorting the 343 biological process (BP) terms in ascending order of adjusted *P* value, we found that the top 25 biological processes (Figure 5) mainly concentrated on aspects of the reactive oxygen species metabolic process and its response to nutrient levels and epithelial cell proliferation. The following processes showed an enriched gene ratio: gland development (GO:0048732, 18.6%), response to nutrient levels (GO:0031667, 18.6%), response to a molecule of bacterial origin (GO:0002237, 15.5%), response to lipopolysaccharide (GO:0032496, 15.2%), response to an antibiotic (GO:0046677, 14.6%), reactive oxygen species metabolic process (GO:0072593, 13.6%), response to oxidative stress (GO:0006979, 16.4%), steroid metabolic process (GO:0008202, 14.2%), cellular response to drug (GO:0035690, 14.9%), epithelial cell proliferation (GO:0050673, 15.8%), response to a steroid hormone (GO:0048545, 14.9%), response to reactive oxygen species (GO:0000302, 11.8%), second-messenger-mediated signaling (GO:0019932, 15.2%), reproductive structure development (GO:0048608, 14.9%), calcium ion transport (GO:0006816, 14.9%), reproductive system development (GO:0061458, 14.9%), regulation of peptide secretion (GO:0002791, 15.8%), response to xenobiotic stimulus (GO:0009410, 12.4%), regulation of membrane potential (GO:0042391, 14.6%), cellular response to oxidative stress (GO:0034599, 12.4%), regulation of epithelial cell proliferation (GO:0050678, 13.6%), divalent metal ion transport (GO:0070838, 15.2%), divalent inorganic cation transport (GO:0072511, 15.2%), negative regulation of response to external stimulus (GO:0032102, 13.3%), and branching morphogenesis of an epithelial tube (GO:0048754, 9.3%). Based on these BP enrichment analyses, we found that the anti-OPF effect of DZ may result from its regulatory effect on the reactive oxygen species metabolic process, response to nutrient levels, and epithelial cell proliferation.

### 3.4. KEGG Pathway Enrichment Analysis

**Figure 6:**
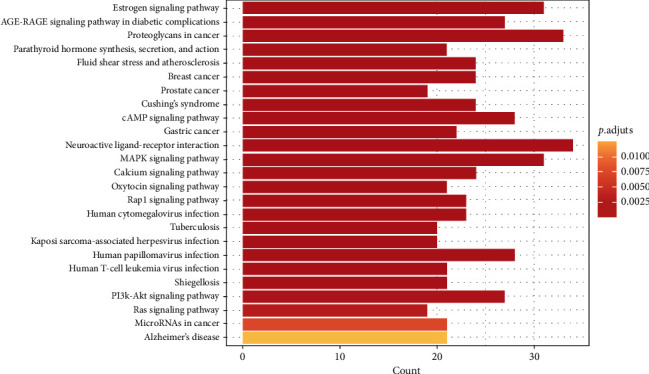
Top 25 pathways of the KEGG enrichment.

**Figure 7:**
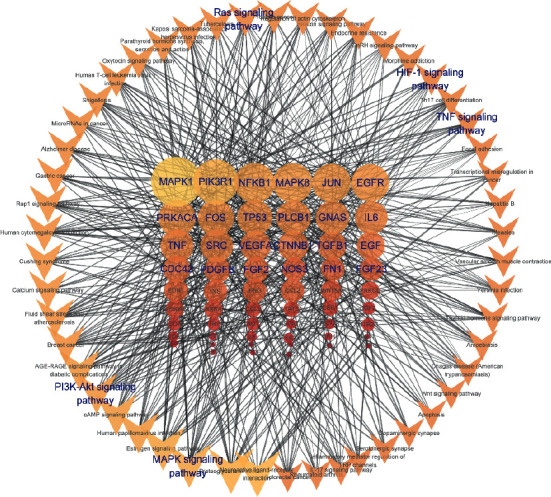
The targets-pathway network of DZ for treating OPF. The circle nodes represent big hub genes and the V-shape nodes represent the top 50 pathways. The nodes' size and colors are dependent on DC value.

We conducted further KEGG pathway enrichment analyses of the 325 overlapping genes to determine the potential therapeutic mechanism of DZ for OPF. Then, we sorted 25 pathways based on the adjusted *P* value (Figure 6). These pathways were the AGE-RAGE signaling pathway in diabetic complications (hsa04933, 9.6%), the estrogen signaling pathway (hsa04915, 11.1%), proteoglycans in cancer (hsa05205, 11.8%), fluid shear stress and atherosclerosis (hsa05418, 8.6%), parathyroid hormone synthesis, secretion, and action (hsa04928, 7.5%), breast cancer (hsa05224, 8.6%), Cushing's syndrome (hsa04934, 8.6%), prostate cancer (hsa05215, 6.8%), the cAMP signaling pathway (hsa04024, 10%), morphine addiction (hsa05032, 6.4%), African trypanosomiasis (hsa05143, 4.3%), the GnRH signaling pathway (hsa04912, 6.4%), endocrine resistance (hsa01522, 6.4%), gastric cancer (hsa05226, 7.9%), neuroactive ligand-receptor interaction (hsa04080, 12.1%), the MAPK signaling pathway (hsa04010, 11.1%), the calcium signaling pathway (hsa04020, 8.6%), the oxytocin signaling pathway (hsa04921, 7.5%), malaria (hsa05144, 4.3%), Th17 cell differentiation (hsa04659, 6.1%), the HIF-1 signaling pathway (hsa04066, 6.1%), colorectal cancer (hsa05210, 5.4%), GnRH secretion (hsa04929, 4.6%), cortisol synthesis and secretion (hsa04927, 4.6%), and the TNF signaling pathway (hsa04668, 6.1%) (Table 2). We also simultaneously constructed the target-pathway network based on the DZ targets enriched in each pathway (Figure 7).

## 4. Discussion

Osteoporosis is becoming a serious health burden due to the aging population. Therefore, prevention of osteoporosis and of osteoporotic fracture has considerable social and economic significance [34]. Drug therapy, such as bisphosphonates and denosumab, is a practical way to treat osteoporosis by inhibiting osteoclast development, formation, and survival [35, 36]. Although these antiresorptive drugs are effective in treating osteoporosis, the long-term use or high dosage of these drugs can cause serious side effects, such as atypical long bone fractures and jaw osteonecrosis [37, 38]. In terms of fracture healing, the use of bisphosphonates remains controversial because callus remodeling is impaired in testing on ovariectomized rats [39]. Furthermore, these antiresorptive drugs cannot rebuild bone that is lost in the progression of osteoporosis, so there is a need to find a method capable of promoting bone building [40].

DZ has been found to alleviate improved osteoblast activity and reverse bone loss in ovariectomized (OVX) mice, which may result from the interaction between its active compounds and related targets. Thus, we investigated the anti-OPF mechanism of DZ through a network pharmacology method. Based on the big hub genes-compounds network (Figure 4), we found that five key compounds, including Civetone, Epiquinidine, quercetin, Gamma-Sitosterol, and Pinosylvin, play dominant roles in this network. Therefore, these active compounds may lay the foundation for the promising anti-OPF effect of DZ.

In the present study, 325 common gene targets of DZ and OPF were identified and 64 hub genes were identified that may play critical roles in this treatment. Some anti-OPF effects of these genes have been confirmed by clinical trials or animal experiments. Chow et al. found that low-magnitude high-frequency vibration stimulation is able to promote fracture healing by positively regulating the impaired innate immune response (lower expression of TNF-*α* and IL-6) and promoting macrophage polarization in delayed osteoporotic fracture healing [41]. Wang et al. suggested that organic gallium can improve osteoporotic fracture healing by positively modulating the OPG/RANKL ratio and inhibiting the expression of TNF-*α* and IL-6 [42]. Zhang et al. found that 17*β*-estradiol, which is used in the clinic to prevent and treat postmenopausal women with osteoporotic fracture, can significantly downregulate ROS production and the expression of TNF-*α* and IL-1*β*, as well as the phosphorylation of ERK2 (MAPK1) [43]. Wang et al. suggested that ESR1 is a susceptibility gene for osteoporotic fracture in postmenopausal Chinese women [44]. De-Ugarte et al. found that miR-320a, which is known to target CTNNB1 of osteoblasts, is overexpressed in osteoporotic fractures [45]. Swanberg et al. found that elderly women who were carriers of the IFNG variant alleles had lower BMD and a lower risk of incident fracture [46]. Based on a large population-based cohort study, Rivadeneira et al. found that ESR2 variants were related to an increased risk of fragility fracture in postmenopausal women [47]. These gene targets play key roles in osteoblast differentiation and osteoclastogenesis during fracture repair. Moreover, the results presented in Figure 4 show that one active compound can interact with multiple genes, one gene with dicompound can interact with multiple genes, and one gene can interact with different compounds, which is in accordance with the modern drug theory of “multi-ingredient, multitarget” [48].

We conducted GO biological process and KEGG enrichment analyses of the overlapping genes to identify their functions. We found 44 (13.6%) genes involved in reactive oxygen species metabolic processes. Reactive oxygen species play key roles in regulating cell proliferation, apoptosis, migration, and differentiation [49], as well as in the regulation of osteoclasts and osteoblasts [50, 51]. EGFR is the overlapping gene between reactive oxygen species (ROS) and the PI3K-Akt signaling pathway (27 genes enriched, 9.6%) based on the results of the GOBP and KEGG pathway enrichment analyses. The PI3K-Akt signaling pathway was first found in tumor cells and proved to regulate numerous cell functions, and it has been proven to be related to the promotion of osteogenesis by upregulating the proliferation and differentiation of bone marrow mesenchymal stem cells [52–54]. Furthermore, activation of the EGFR-Akt-mTOR pathway can effectively protect osteoblasts against dexamethasone, which increases the level of ROS production in osteoblastic cells [55]. MAPK1 is a common gene in reactive oxygen species, the PI3K-Akt signaling pathway, the MAPK signaling pathway (31 genes enriched, 11.1%), and the TNF signaling pathway. MAPKs play important roles in cell proliferation, differentiation, and apoptosis, as well as in regulating inflammation [56]. ROS can lead to serious periodontal tissue destruction by promoting osteoclastic bone resorption, which is closely related to the protein expression of MAPKs and NF-*κ*B [57]. Srinivasa et al. found that the facilitation of ROS on osteoclast differentiation and resorption can be reversed by inhibiting the NF-*κ*B and calcineurin-NFAT pathways [58]. The activation of MAPKs also plays a key role in osteoclastogenesis induced by RANKL. However, these ROS-directed upregulations of MAPK and NF-*κ*B signaling can be attenuated by strengthening the nuclear factor-erythroid 2-related factor 2 [59]. Therefore, the anti-OPF effect of DZ may depend on the regulatory effect on ROS through these key genes and pathways.

Forty-five (13.9%) genes were involved in the regulation of the inflammatory response. In vitro and in vivo studies have shown that the proinflammatory cytokines IL-6 and TNF-*α* are involved in the pathogenesis of osteoporosis and influence bone metabolism [60, 61]. Several studies have also found that high expression of inflammatory markers is closely related to increased bone loss [62–64] and that these markers are also risk factors for incidents of nontraumatic fractures. Women with increased expression of inflammatory markers have a 3-fold risk of hip fractures [65]. IL-6 and TNF-*α* are the overlapping genes between the regulation of inflammatory response (17 genes enriched, 6.1%) and the TNF signaling pathway (45 genes enriched, 13.9%) based on the results of the BP and KEGG pathway enrichment analyses. Ali Aydin et al. found higher expression levels of TNF- *α* and IL-6 in OVX model rats, and these cytokines reached their apex when a fracture occurred [66]. Moreover, these cytokines also increase the production of oxygen radicals, which can enhance osteoclastic activity while decreasing osteoblastic activity [67, 68]. Therefore, the anti-OPF effect of DZ may depend on the regulation of the inflammatory response through these key genes and pathways.

## 5. Conclusion

By utilizing network pharmacology, we investigated the potential targets of DZ and the underlying mechanism of its anti-OPF effects, which may be based on the regulation of ROS and the inflammatory response. According to the KEGG pathway enrichment results, we found that the PI3K-Akt signaling pathway, MAPK signaling pathway, and TNF signaling pathway may be the main pathways in treating OPF. Thus, we believe that the anti-OPF effect of DZ is mainly based on its direct or indirect regulation of the abovementioned potential targets and pathways and that DZ provides promising directions for future research, which is essential to reveal its exact regulatory mechanisms.

## Figures and Tables

**Table 1:** Information of 64 hub targets.

UniProt ID	Gene symbol	Description	Degree
Q499G7	MAPK1	Mitogen-activated protein kinase 1	41
P12931	SRC	SRC proto-oncogene, non–receptor tyrosine kinase	39
P27986	PIK3R1	Phosphoinositide-3-kinase regulatory subunit 1	36
P15692	VEGFA	Vascular endothelial growth factor A	31
Q53GA5	TP53	Tumor protein p53	29
Q504U8	EGFR	Epidermal growth factor receptor	29
P05412	JUN	Jun proto-oncogene	29
P01019	AGT	Angiotensinogen	29
Q75MH2	IL6	Interleukin 6	28
P01133	EGF	Epidermal growth factor	27
P45983	MAPK8	Mitogen-activated protein kinase 8	26
Q6FG41	FOS	Fos proto-oncogene	25
P00734	F2	Coagulation factor II, thrombin	25
Q9UQS6	FN1	Fibronectin 1	24
Q6FH53	EDN1	Endothelin 1	24
Q5STB3	TNF	Tumor necrosis factor	24
P02775	PPBP	Pro-platelet basic protein	24
Q9UBT1	ESR1	Estrogen receptor 1	23
Q15788	NCOA1	Nuclear receptor coactivator 1	23
P35222	CTNNB1	Catenin beta 1	23
P04150	NR3C1	Nuclear receptor subfamily 3 group C member 1	23
P02768	ALB	Albumin	22
Q6P3U7	RXRA	Retinoid *X* receptor alpha	21
P22681	CBL	Cbl proto-oncogene	21
P01308	INS	Insulin	20
P17612	PRKACA	Protein kinase cAMP-activated catalytic subunit alpha	18
P02765	AHSG	Alpha 2-HS glycoprotein	18
P01137	TGFB1	Transforming growth factor beta 1	18
V9HW22	HSPA8	Heat shock protein family A (Hsp70) member 8	17
Q9NUA2	AR	Androgen receptor	17
Q9GZV9	FGF23	Fibroblast growth factor 23	17
Q5JWF2	GNAS	GNAS complex locus	16
P19838	NFKB1	Nuclear factor kappa B subunit 1	16
Q14393	GAS6	Growth arrest specific 6	15
Q05655	PRKCD	Protein kinase C delta	15
P30518	AVPR2	Arginine vasopressin receptor 2	15
P02647	APOA1	Apolipoprotein A1	15
P01127	PDGFB	Platelet-derived growth factor subunit B	15
Q6PK50	HSP90AB1	Heat shock protein 90 alpha family class B member 1	14
Q5FC01	IL4	Interleukin 4	14
P0DMV9	HSPA1A	Heat shock protein family A (Hsp70) member 1A	14
P01344	IGF2	Insulin-like growth factor 2	14
Q96EB6	SIRT1	Sirtuin 1	13
Q4W448	PPARG	Peroxisome proliferator activated receptor gamma	13
Q07869	PPARA	Peroxisome proliferator activated receptor alpha	13
P60953	CDC42	Cell division cycle 42	13
P06401	PGR	Progesterone receptor	13
P01579	IFNG	Interferon gamma	13
P01034	CST3	Cystatin C	13
Q9Y6Q9	NCOA3	Nuclear receptor coactivator 3	12
Q9Y494	TAC1	Tachykinin precursor 1	12
Q9NQ66	PLCB1	Phospholipase C beta 1	12
P29474	NOS3	Nitric oxide synthase 3	12
P13501	CCL5	C-C motif chemokine ligand 5	12
P09038	FGF2	Fibroblast growth factor 2	12
Q92731	ESR2	Estrogen receptor 2	11
Q59GY7	STAT5A	Signal transducer and activator of transcription 5A	11
P41221	WNT5A	Wnt family member 5A	11
P28335	HTR2C	5-Hydroxytryptamine receptor 2C	11
P28223	HTR2A	5-Hydroxytryptamine receptor 2A	11
P13500	CCL2	C-C motif chemokine ligand 2	11
P06850	CRH	Corticotropin releasing hormone	11
P02649	APOE	Apolipoprotein E	11
P00746	CFD	Complement factor D	11

**Table 2:** Information of 25 pathways.

ID	Description	*P*. adjust	Count
hsa04933	AGE-RAGE signaling pathway in diabetic complications	4.29750564649423e-15	27
hsa04915	Estrogen signaling pathway	4.29750564649423e-15	31
hsa05205	Proteoglycans in cancer	7.02978144058047e-12	33
hsa05418	Fluid shear stress and atherosclerosis	3.50540676797616e-09	24
hsa04928	Parathyroid hormone synthesis, secretion, and action	3.50540676797616e-09	21
hsa05224	Breast cancer	8.65129662473866e-09	24
hsa04934	Cushing syndrome	2.25061036469472e-08	24
hsa05215	Prostate cancer	2.25061036469472e-08	19
hsa04024	cAMP signaling pathway	4.25537215261066e-08	28
hsa05032	Morphine addiction	4.35290458036493e-08	18
hsa05143	African trypanosomiasis	5.25038311520481e-08	12
hsa04912	GnRH signaling pathway	5.25038311520481e-08	18
hsa01522	Endocrine resistance	1.16942061208876e-07	18
hsa05226	Gastric cancer	1.53049773606446e-07	22
hsa04080	Neuroactive ligand-receptor interaction	3.92997114777747e-07	34
hsa04010	MAPK signaling pathway	4.75554213381079e-07	31
hsa04020	Calcium signaling pathway	8.10557748077706e-07	24
hsa04921	Oxytocin signaling pathway	1.09530352609987e-06	21
hsa05144	Malaria	1.3497422679709e-06	12
hsa04659	Th17 cell differentiation	1.90497387620456e-06	17
hsa04066	HIF-1 signaling pathway	2.39525818923615e-06	17
hsa05210	Colorectal cancer	2.67545458381123e-06	15
hsa04929	GnRH secretion	2.67545458381123e-06	13
hsa04927	Cortisol synthesis and secretion	3.01425725456316e-06	13
hsa04668	TNF signaling pathway	3.01425725456316e-06	17

## Data Availability

All our main data used to support the findings of this study have been deposited in the Figshare repository (https://doi.org/10.6084/m9.figshare.12152607.v1).

## Abbreviations

DZ: Du Zhong
OPF: Osteoporotic fractures
TCM: Traditional Chinese medicine
TCMSP: Traditional Chinese Medicine System Pharmacology Database
OB: Oral bioavailability
DL: Drug-likeness
CTD: Comparative Toxicogenomics Database
OMIM: Online Mendelian Inheritance in Man
HPO: Human Phenotype Ontology
PPI: Protein-protein interaction
KEGG: Kyoto Encyclopedia of Genes and Genomes
GO: Gene Ontology
BP: Biological process
OVX: Ovariectomized
ROS: Reactive oxygen species.
